# Intestinal parasites co-infection among tuberculosis patients in Ethiopia: a systematic review and meta-analysis

**DOI:** 10.1186/s12879-020-05237-7

**Published:** 2020-07-14

**Authors:** Ayinalem Alemu, Zebenay Workneh Bitew, Teshager Worku

**Affiliations:** 1 Ethipian Public Health Institute, Addis Ababa, Ethiopia; 2 Department of Pediatric Nursing, School of Nursing, St Paul’s Hospital Millennium Medical College, Addis Ababa, Ethiopia; 3 School of Nursing and Midwifery, College of Health and Medical Sciences, Haramaya University, Harar, Ethiopia

**Keywords:** Helminthes, *Hook worm*, Multiple parasitic infection, *Ascaris Lumbricoides*

## Abstract

**Background:**

Tuberculosis and intestinal parasites are mostly affecting poor people. They are in a vicious since one is the risk factor for the other. However, the comprehensive report on the burden and co-incidence of intestinal parasites and tuberculosis in Ethiopia is scant. This systematic review and meta-analysis aimed to provide abridge conclusive evidence on the intestinal parasite-tuberculosis co-infection in Ethiopia.

**Methods:**

A total of 414 articles published in English were searched from both electronic databases (CINAHL, DOAJ, Embase, Emcare, Medline, ProQuest, and PubMed, Science Direct, and Web of Science) and other sources. The qualities of the included studies were assessed using the Joanna Briggs Institute Critical Appraisal tools and the publication bias was measured using the funnel plot and Eggers regression test. Comprehensive meta-analysis (CMA) Version 3.3.07 and Review Manager software were used to estimate pooled prevalence and associations of intestinal parasites and tuberculosis infection.

**Results:**

Eleven articles with a total of 3158 tuberculosis patients included in the analysis based on the eligibility criteria. The estimated pooled prevalence of intestinal parasites co-infection was 33% (95% CI: 23.3, 44.3) using the random-effects model. The most common intestinal parasites were *Ascaris lumbricoides* 10.5% (95% CI: 6.0, 17.5), *Hookworm* 9.5% (95% CI: 6.10, 14.4), *Giardia lamblia* 5.7% (95% CI: 2.90, 10.9) and *Strongyloides sterocoralis* 5.6% (95% CI: 3.3, 9.5). The odds of intestinal parasites infection was higher among tuberculosis patients compared to tuberculosis free individuals (OR = 1.76; 95% CI: 1.17, 2.63). A significant difference was observed among TB patients for infection with intestinal helminths (OR = 2.01; 95% CI: 1.07, 3.80) but not for intestinal protozoans when compared with their counterparts. The odds of multiple parasitic infections was higher among tuberculosis patients (OR = 2.59, 95% CI: 1.90, 3.55) compared to tuberculosis free individuals. However, intestinal parasites co-infection was not associated with *HIV* status among tuberculosis patients (OR = 0.97; 95% CI: 0.71, 1.32).

**Conclusion:**

One-third of tuberculosis patients are co-infected with one or more intestinal parasites, and they are more likely to be infected with intestinal helminths and multiple intestinal parasitic infections compared to TB free individuals. We recommend routine screening of tuberculosis patients for intestinal parasites. The effect of mass deworming on tuberculosis incidence would be important to be considered in future researches.

**Trial registration:**

Registered on PROSPERO with reference number ID: CRD42019135350.

## Background

Tuberculosis (TB) is the leading cause of death among infectious diseases. Globally, TB causes 10.0 million illnesses and 1,451,000 deaths in 2018. Most of these cases were reported from African and Asian countries [1]. Even though TB affects all countries, the problem is worse in developing countries where the prevalence of intestinal parasites and human immunodeficiency virus (HIV) are higher [2]. Tuberculosis and intestinal parasites substantially share similar geographical settings [3, 4].

Intestinal parasites affect many people worldwide, but higher in developing countries [5]. Different studies reported the geographical overlap of both infections, especially in Sub-Saharan African countries [6]. Intestinal parasites are reported as risk factors to develop TB [3, 5]. Specifically, helminths have immune-modulatory mechanisms to live in a host for years [7, 8]. This modulation shifts the immune system to sub divert to T-helper cell (Th) 2 cytokines and causes the human host to be susceptible to *Mycobacterium tuberculosis* infection [3, 4, 7]. Enhanced Th1 immune response is important to protect against TB, while reduced Th1 cytokines and enhanced Th2 and T-regulatory (Treg) are associated with TB susceptibility [9]. The risk of TB is higher among helminths infected individuals through affecting the host immunity to TB [3, 10]. Tuberculosis patients harbor more intestinal parasites compared to TB free individuals [3, 11, 12]. However, there are controversies on the relation of intestinal parasites and TB infection. In a study done by Neto et al. [5] in Brazil, the findings neither show an association between helminthic infection and a favorable TB outcome, nor between parasitism and tuberculin skin test (TST) response.

Ethiopia is among countries being seriously affected by both tuberculosis and intestinal parasites infections [12]. There were 151 estimated TB cases per 100,000 populations, where the country is included under high TB burden countries [1]. Likewise, intestinal parasites are causing a significant number of infections in the country [13]. Studies conducted in different parts of Ethiopia showed a higher prevalence of intestinal parasitic co-infection among TB patients as compared to the TB free individuals [3, 11, 12]. A 70.9% prevalence of intestinal helminthic co-infection among TB patients is reported from Northwest Ethiopia [3]. Some studies reported that there was a higher prevalence of intestinal parasite infection among TB patients compared to TB free individuals [3, 11, 12, 14], while others reported no association between intestinal parasitic infection and TB [4, 15–17]. Even though there are several usable studies with apparent variability in the country, to our knowledge, there is no conclusive evidence of tuberculosis and intestinal parasites co-infection. Thus, this systematic review and meta-analysis aimed to provide a bridge conclusive evidence on the intestinal parasite-tuberculosis co-infection in Ethiopia.

## Methods

### Protocol and registration

The protocol for the systematic review and meta-analysis has been registered in the International Prospective Register of systematic reviews (PROSPERO) ID: CRD42019135350. The methodology of this systematic review and meta-analysis was developed following the Preferred Reporting Items for Systematic Reviews and Meta-Analyses (*PRISMA****)*** reporting checklist.

### Inclusion criteria

We reviewed studies based on PICOS (participants, interventions, comparison, outcome, and study setting) criteria. Original studies (cross-sectional and case-control studies) conducted from 2000 to 2019 published in the English language that showed intestinal parasitic co-infection among TB patients in Ethiopia were included. Studies that confirmed TB either bacteriologically or by pathology or through x-ray findings were considered.

### Exclusion criteria

Articles without fill documents, articles conducted on Ethiopians reside out of Ethiopia were excluded. Commentaries, case reports, case series, and proceedings were excluded.

### Information sources and searching strategy

The articles were systematically searched from the available electronic database (CINAHL, DOAJ, EMBASE, Emcare, Medline, ProQuest, PubMed, Science Direct, and Web of Science) and other grey literature sources. Two researchers (Teshager Worku (TW) and Zebenay Workneh Bitew (ZWB)) independently searched articles from the identified database. The articles were searched using the search string taken from research questions and applied to each database as required. The keywords used for constructing search string were tuberculosis OR TB, parasite, helminths, and Ethiopia. The Boolean operators; OR and AND were applied while searching. The search string applied for Medline (Ovid) database was ((Intestinal disease, parasitic/ OR Intestinal parasites.mp.) AND (Tuberculosis/ OR Tuberculosis.mp.) AND (Ethiopia.mp. OR Ethiopia/)) (See Additional file 1).

### Study selection procedure

All articles extracted from different sources were exported to EndNote X8 citation manager, and duplicates were removed. The abstract screening performed before the full-text screening and, consequently, only those which passed the abstract screening were fully appraised. Two authors (Ayinalem Alemu (AA) and ZWB) screened the title and abstracts of the studies with predefined inclusion criteria independently. Two authors (AA and TW) also independently collect full texts and evaluate the eligibility of them for final inclusion by considering study subjects, language, study designs, quality, and outcome. The full texts of eligible articles were assessed for quality using Joanna Briggs Institute Critical Appraisal (JBI) tools [18] (See Additional file 2).

### Data extraction

Included studies’ characteristics (Author, year of publication, study area/region, study design, sample size, TB screening method, the name and number of intestinal parasites, subgroup data containing outcomes of intestinal parasites among TB patients and controls, multiple parasitic infections and *HIV* status of TB patients) were extracted in a template prepared using Microsoft word (2016) (Tables 1, 2 & 3). The quantitative data for meta-analysis from each study were extracted and stored in Microsoft excel (2016). The two authors (ZWB and AA) independently extracted data from all of the included studies.

**Table 1:** Characteristics of individual studies on intestinal parasites co-infection among tuberculosis patients in Ethiopia; included in the current systematic review and meta-analysis

Author	Publication year	Place of study	Study design	Study setting	Clinics used as sources	Study period	Sampling method	Methods used to diagnose parasites				TB pts.	Infected with intestinal parasites	Prevalence (%)	Quality score
								Direct microscopy	Formal ether concentration	Modified Ziehl Nielsen	Kato-Katz				
Alemu et al [11]	2019	Addis Ababa	Case-control	Health facility	Health center	Jan 2017 to Jan 2018	Consecutive	Yes	Yes	Yes	No	91	20	22.0	Medium
Hailu et al [12]	20 15	Woldia	Case-control	Health facility	Hospital and health center	November 2010 to June 2011	Consecutive	Yes	Yes	Yes	No	100	49	49.0	High
Alemu et al [19]	2017	Arba Minch	Cross-sectional	Health facility	Hospital and health center	January to August 2016.	Consecutive	Yes	Yes	No	No	213	56	26.3	High
Kassu et al [20]	2007	Northwest Ethiopia	Cross-sectional	Health facility	Hospital	January 2003 and August 2003	Consecutive	Yes	Yes	No	No	257	104	40.5	Medium
Elias et al. [3]	2006	Northwest Ethiopia	Case-control	Health facility	Hospital	October 1999 and January 2002	Consecutive	Yes	Yes	No	No	230	163	70.9	High
Abate et al [4]	2012	Northwest Ethiopia	Case-control	Health facility	Hospital and health center	–	Consecutive	Yes	No	No	Yes	112	32	28.6	Medium
Alemayehu et al [15]	2014	Northwest Ethiopia	Cross-sectional	Health facility	Hospital and health center	March 2008–May 2008	Consecutive	Yes	Yes	No	No	72	24	33.3	Medium
Tegegne et al [17]	2018	Northwest Ethiopia	Cross-sectional	Health facility	Hospital	March to May 2017	Systematic random sampling	Yes	Yes	No	No	43	5	11.6	Medium
Gashaw et al [21]	2019	Northeast Ethiopia	Cross-sectional	Health facility	Hospital and health center	April 2015 to January 2017	Consecutive	Yes	Yes	No	Yes	259	28	10.8	Medium
Ramose et al [16]	2009	Gambo	Cross-sectional	Health facility	Hospital	September 2002 to October 2003.	Consecutive	Yes	No	No	No	100	32	32.0	Medium
Feleke et al [14]	2019	Amhara region	Comparative cross-sectional	Community-based	Health center	July 2015–May 2018	Systematic random sampling	No	No	Yes	Yes	1681	893	53.1	Medium

**Table 2:** Prevalence of each identified intestinal parasites among tuberculosis patients in Ethiopia

Name of intestinal parasite	Number of studies	Prevalence (95% CI)	Heterogeneity tests			
			Q test	df	I^**2**^	***p-value***
*A.lumbricoides*	11	0.105 (0.060–0.175)	21.9	10	77%	< 0.001
*H.worm*	9	0.095 (0.061–0.144)	77.6	8	90%	< 0.001
*G.lamblia*	7	0.057 (0.029–0.109)	38.3	6	84%	< 0.001
*S.sterocolaris*	8	0.056 (0.033–0.095)	56	7	87.5%	< 0.001
*S.mansoni*	6	0.046 (0.034–0.061)	1	5	0.0%	0.961
*C.parvum*	2	0.043 (0.017–0.105)	1.6	1	36.8%	0.206
*E.histolytica*	6	0.038 (0.018–0.079)	21.9	5	77%	0.001
*T.trichuria*	9	0.031 (0.016–0.059)	34	8	76.6%	< 0.001
*I.belli*	1	0.03(−0.003–0.063)	–	–	–	–
*Taenia spp.*	3	0.06 (0.007–0.039)	2.8	2	27.6%	0.251
*E.vermiculais*	3	0.012 (0.005–0.026)	1.8	2	0.0%	0.405
*H. nana*	3	0.011 (0.003–0.038)	8.8	2	65.8%	0.032

*df* degree of freedom

**Table 3:** Intestinal parasitic infection among tuberculosis patients and tuberculosis free individuals in Ethiopia

Group variable	Number of studies	TB patients		TB free individuals		OR(95% CI)	Heterogeneity tests			
		Number	%	Number	%		X^**2**^	df	I^**2**^	***p-value***
Total intestinal parasites	8	1218	49.1%	2076	37.4%	1.76 (1.17–2.63)	50.95	7	86%	0.006
Intestinal protozoans	4	64	17.6%	129	10.0%	1.66 (0.94–2.93)	6.26	3	52%	0.08
Intestinal helminthes	7	277	37.0%	486	22.2%	2.01 (1.07–3.80)	43.18	6	86%	0.03
Multiple parasite infection	4	87	17.3%	116	6.8%	2.59 (1.90–3.55)	4.47	3	33%	< 0.001

*X*^*2*^ Chi-square, *df* degree of freedom

### Data items

Population: Tuberculosis patients.

Intervention: Not available.

Comparators: Tuberculosis free individuals.

Outcomes: Intestinal parasite-tuberculosis co-infection, Odds ratio.

Study type: Observational studies.

### Risk of bias (quality) assessment of individual studies

The methodological reputability and quality of the findings of the included studies were critically evaluated using the quality assessment tool for observational studies (cross-sectional and case-control studies) developed by the Joanna Briggs Institute (JBI) [22]. By using the JBI checklist, for cross-sectional studies, eight variables are used to score out of 100% and each variable scored from 12.5% and then turned into 100%. Case-control studies 10 variables are scored out of 100% where one variable scored out of 10%. Quality score is graded as low if < 60%, medium if 60–80% and high if > 80%. To ensure quality, we tried to search for studies using a comprehensive strategy (electronic databases, and manual search); included published and/or unpublished studies. Studies were screened by two independent authors (AA & TW) using clear objective eligibility criteria to minimize bias. Publication bias was explored using visual inspection of the funnel plot [23]. Besides, Egger’s regression test [24] was carried out to check the statistical symmetry of the funnel plot.

### Data synthesis and statistical analysis

The estimated pooled prevalence of intestinal parasites co-infection among TB patients with its 95% Confidence Interval (CI) by assuming the true effect size varies between studies was determined. The pooled prevalence was determined as the ratio of numbers of TB patients who were co-infected with intestinal parasites to the total sample size (total TB patients). The data was presented on a forest plot. The heterogeneity in the prevalence of the different studies was assessed using I^2^. The specific analysis was also done based on the outcomes of intestinal parasites, types of parasites identified, multiple parasitic infections, and *HIV* status. Comprehensive meta-analysis (CMA) Version 3.3.07 was used to estimate the pooled prevalence of intestinal parasites co-infection and the pooled prevalence of each parasite TB co-infections. Review Manager Software (version 5.3) [25] was used to analyze the specific intestinal parasite TB co-infection and associations.

## Results

### Study characteristics

**Fig. 1:**
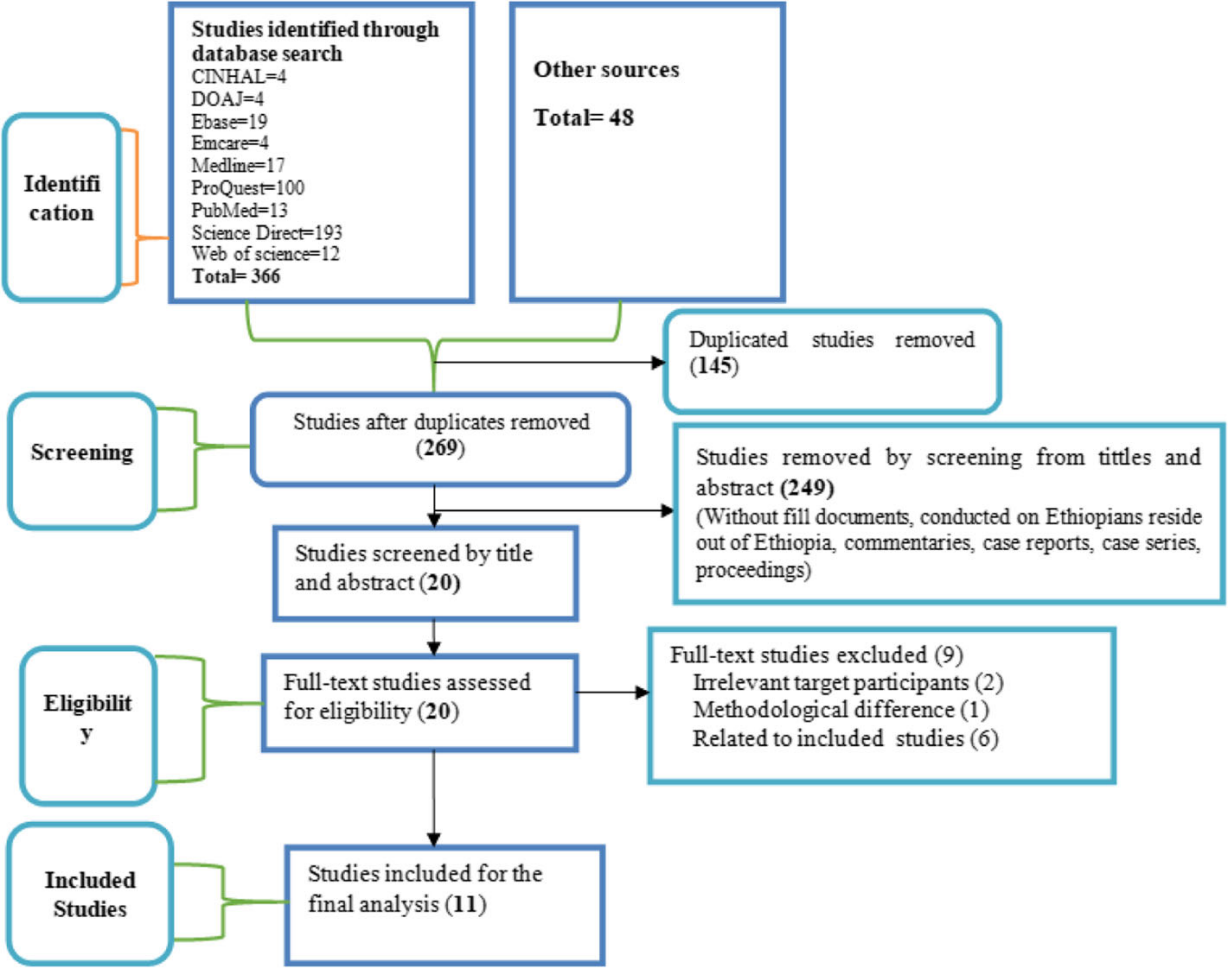
Flowchart diagram describing selection of studies for the systematic review and meta-analysis on intestinal parasites co-infection among tuberculosis patients in Ethiopia

**Fig. 2:**
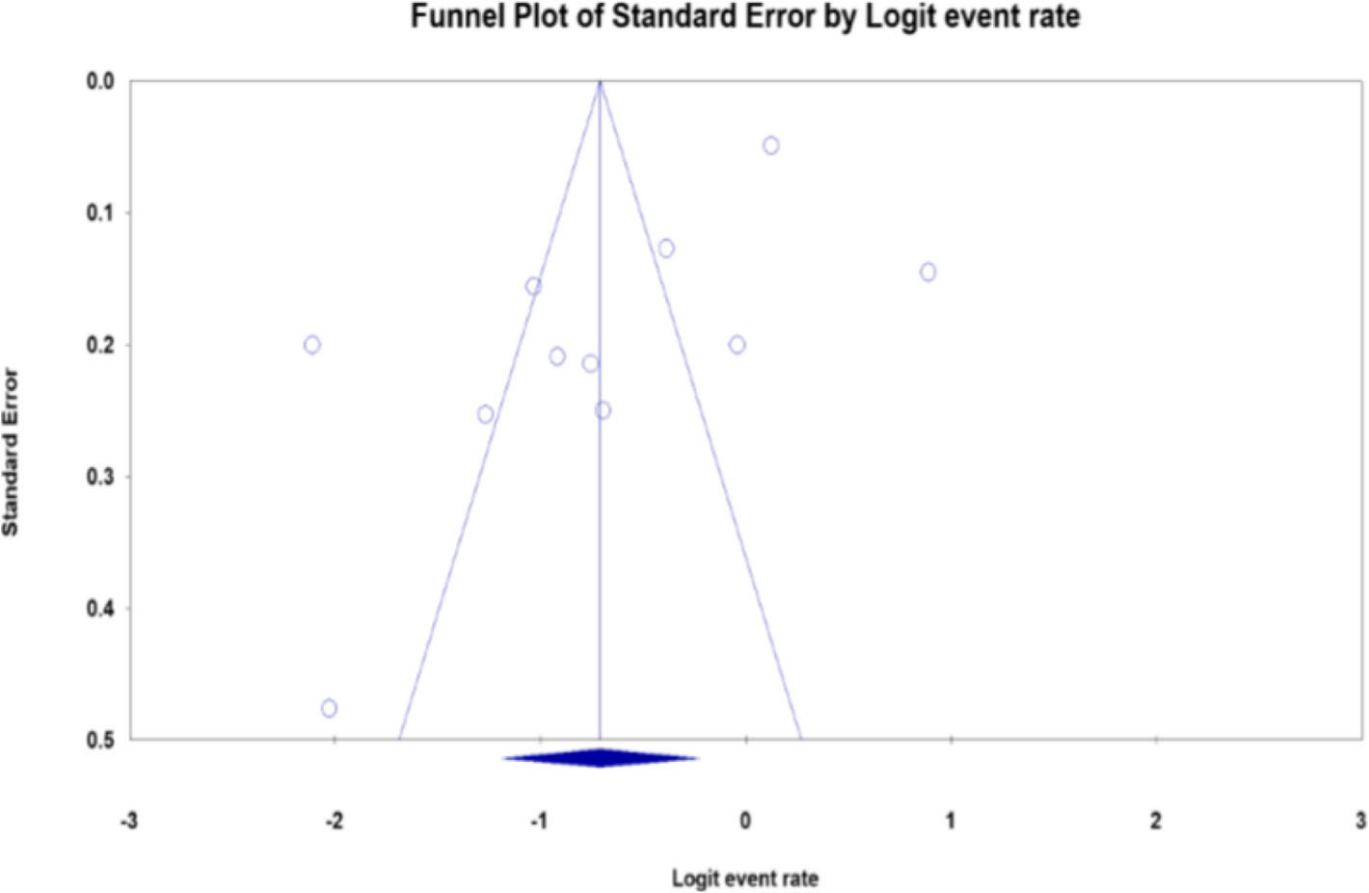
Funnel plot for pooled prevalence of intestinal parasites co-infection among tuberculosis patients in Ethiopia

From the available scientific database and other sources**,** 414 studies were searched. Of this, 145 duplicate studies were removed. Two hundred sixty-nine studies were screened by title and abstract. Finally, only 20 papers were found to be eligible for full-text evaluation, and 11 studies [3, 4, 11, 12, 14–17, 19–21] that fulfilled the eligibility criteria were included in the final analysis (Table 1, Fig. 1). The remaining nine studies were excluded from the analysis based on the irrelevant target population [26, 27], the methodological difference [28], and being related to the included studies [9, 29–33] (Fig. 1). From the included studies, four of them were case-control studies while the others were cross-sectional studies. The sample size in each included study ranged from 43 to 1681 TB patients. However, except for a study done by Feleke et al. [14] that screened 1681 TB patients for intestinal parasites, the sample size for each of the remaining 10 studies was below 300. To diagnose TB, most of the studies used smear microscopy while one study from Addis Ababa and the other study from northeast Ethiopia used *Mycobacterial* culture. Controls or comparative groups were classified as TB free individuals through the use of diagnostic methods used to confirm TB. Likewise, laboratory diagnostic methods used to examine intestinal parasites among TB patients and TB free individuals were assessed. Accordingly, different combinations of stool examination methods were used. Ten [3, 4, 11, 12, 15–17, 19–21], nine [3, 7, 11, 12, 14, 15, 17, 19, 21], three [4, 14, 21] and two studies [11, 12] used direct saline examination, formol ether concentration, Kato-Katz and modified Ziehl Neelsen methods respectively. Two studies that used modified Ziehl Nelson staining method reported coccidian parasites co-infection among TB patients. Five studies [3, 4, 12, 19, 20] assessed the *HIV* serostatus of TB patients. We pooled and compared intestinal parasites co-infection rates between 335 *HIV* positive TB patients and 538 *HIV* negative TB patients. The heterogeneity of included studies was analyzed by I^2^ static and a high level of heterogeneity was found. To minimize heterogeneity, a sensitivity analysis was done and fixed and random effect models were used interchangeably. The publication bias was assessed using a funnel plot and Egger’s test (*p* < 0.05). The funnel plot was asymmetrical and Egger’s test indicted there was publication bias (*P* = 0.028) (Fig. 2).

### The pooled prevalence of intestinal parasites among tuberculosis patients

**Fig. 3:**
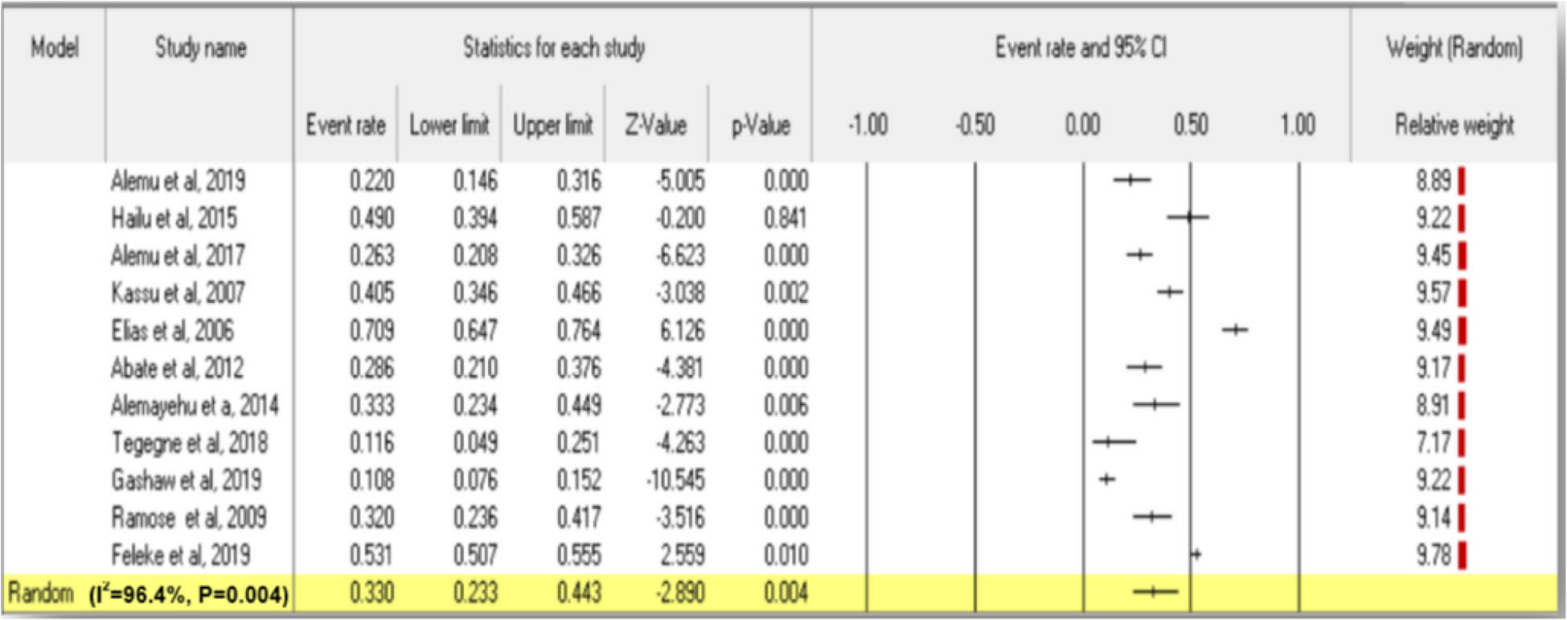
Forest plot for pooled prevalence of intestinal parasites co-infection among tuberculosis patients in Ethiopia

A total of 3158 TB patients were assessed for intestinal parasites co-infection. All individual studies reported intestinal parasites co-infection prevalence among TB patients beyond 10%, and except for two studies [17, 21], it was greater than 22%. The highest prevalence (70.9%) was reported from Northwest Ethiopia [20], while the least prevalence (10.8%) was reported from northeast Ethiopia [21]. The overall estimated pooled prevalence of intestinal parasites co-infection among TB patients using a random-effects model was 33% (95% CI: 23.3, 44.3) (I^2^ = 96.4%, *P =* 0.004) (Fig. 3).

Twelve types of intestinal parasites were reported from the stool examination of TB patients. All 11 studies reported the detection of *A.lumbricoides* among TB patients. Secondly, nine studies [3, 4, 15–17, 19–21] reported the detection of *H.worm* and similarly nine [3, 4, 11, 12, 15, 16, 19–21] reported *T.trichuria*. Likewise, *S.sterocolaris* was reported by eight [3, 4, 12, 14–16, 19, 20] studies. Among intestinal protozoans, *G.lamblia* was repeatedly reported by seven [11, 12, 15, 16, 19–21] studies. *C.parvum and I.belli* were the least reported in terms of the number of studies reported the parasites where *C.parvum* was reported from two studies [11, 12] and only one study [12] reported the detection of *I.belli* (Table 2). According to our meta-analysis result, the most common parasites were *A.lumbricoides* 10.5% (95% CI: 6.0, 17.5), *H.worm* 9.5% (95% CI: 6.10, 14.4), *G.lamblia* 5.7% (95% CI: 2.90, 10.9) and *S.sterocoralis* 6% (95% CI: 3.3, 9.5) (Supplementary figures).

### Associations of intestinal parasites and tuberculosis infection

**Fig. 4:**
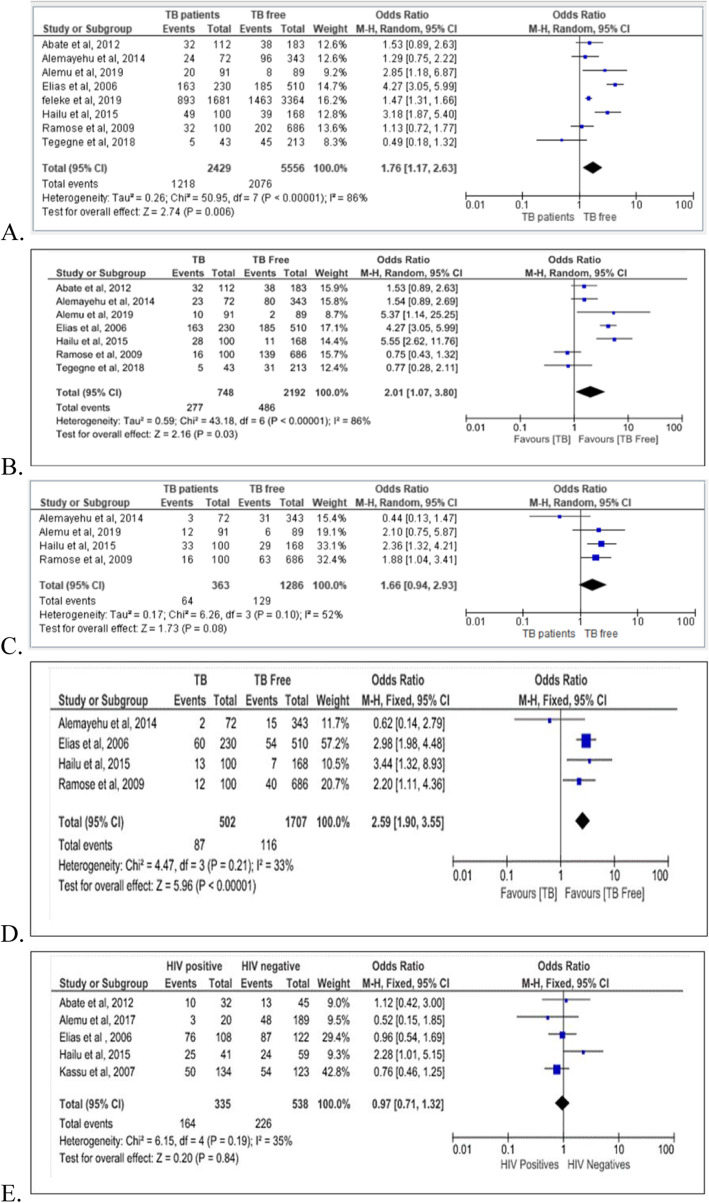
Forest plot for comparison between tuberculosis patients and tuberculosis free individuals in Ethiopia. **a** Intestinal parasitic infection. **b** Intestinal helminthes infection. **c** Intestinal protozoan infection. **d** Multiple intestinal parasitic infections. **e** Intestinal parasitic infection among tuberculosis patients by their *HIV* sero-positivity status

Intestinal parasites co-infection among 2429 TB patients and 5556 TB free individuals were compared. Eight studies [3, 4, 11, 12, 14–17] reported intestinal parasites co-infection among these two groups. Based on the pooled analysis, TB patients had 1.76 times the odds to harbor more intestinal parasites compared to TB free individuals (OR = 1.76; 95% CI: 1.17, 2.63) (Table 3, Fig. 4). Intestinal parasites were categorized into two groups namely; intestinal helminths and intestinal protozoans. A statistically significant difference was observed among TB patients and TB free individuals for helminthic infections but not for protozoans. Tuberculosis patients had double risk to be infected with intestinal helminths compared to TB free controls (OR = 2.01; 95% CI: 1.07, 3.80) (Table 3, Fig. 4). Multiple (two or more) intestinal parasitic infections were frequently detected from TB patients compared to TB free individuals. Tuberculosis patients had 2.59 times the odds to harbor multiple intestinal parasites compared to TB free individuals (OR = 2.59; 95% CI: 1.90, 3.55) (Fig. 4). There was no statistically significant difference in parasitic TB co-infection for *HIV* positive TB patients compared with *HIV* negative TB patients (Fig. 4).

## Discussion

To our knowledge, this study is the first comprehensive systematic review and meta-analysis on intestinal parasites co-infection among tuberculosis patients in Ethiopia where both infections are the major public health problems. After intense searching, 11 studies [3, 4, 11, 12, 14–17, 19–21] were included in the analysis. Based on the pooled prevalence analysis, one-third of TB patients (33.0%) were co-infected with intestinal parasites. This shows that considerable proportions of TB patients were co-infected with intestinal parasites and this co-infection is a public health problem in the country. When compared to individual studies conducted in other countries, comparable results were reported from Tanzania [6] and Iran [34]. Lower prevalence was reported from China [35] and the higher prevalence was reported from Brazil [10]. However, individual studies included in this meta-analysis reported a higher co-infection rate [3, 12, 14]. In the current systematic review and meta-analysis study, heterogeneity was detected (Fig. 2). This might be happening due to the methodological difference where the higher co-infection rate was reported from case-control studies [3, 12, 20]. However, all studies were health facility-based studies that represented TB patients that visited health facilities for health care, and in most of the studies, TB patients were recruited with consecutive sampling methods.

Among the 12 types of intestinal parasites identified, *A.lumbricoides* and *H.worm* were found to be the most frequently reported intestinal parasites. Likewise, studies from Tanzania [6] and Egypt [36] reported supportive findings. Among intestinal protozoans, *G.lamblia* was reported frequently compared with other intestinal protozoans. Two studies [11, 12] used a modified Ziehl Neelsen staining method to identify coccidian parasites, and both reported *C.parvum.* However, *I.belli* was reported only from one study [12]. Hailu et al [12] reported *C.parvum* and *I.belli* co-infections from *HIV* positive TB patients that might worsen the problem.

larized immune response hasThere is a statistically significant difference in intestinal parasitic infection among TB patients and TB free individuals (OR = 1.76; 95% CI: 1.17, 2.63). TB patients had 1.76 times the odds to harbor more intestinal parasites compared to TB free individuals. The pooled analysis showed that a statistically significant difference infection rate among TB patients and TB free individuals observed for intestinal helminths (OR = 2.01; 95% CI: 1.07, 3.80) but not for intestinal protozoans (OR = 1.66; 95% CI: 0.94, 2.93). This supported scientific evidence that helminthic infections had an immune modulation mechanism that enabled them to escape the immune system and live inside a host for many years [7]. It was reported that helminths cause immune activation with biased Th2 responses and down-regulated Th1 and Cytolytic T lymphocytes activity that might make individuals susceptible to infections that are limited by Th1 response [3, 4, 7]. In sub-Saharan Africa, a dominant Th2 po been reported and suggested to increase susceptibility to *Mycobacterium tuberculosis* [3, 4].

Multiple parasitic infections and repeated infections are common in areas where parasites are highly prevalent, and this might make people susceptible to other infections such as tuberculosis. Studies reported that individuals with high worm burden or multiple infections had an increased risk of developing TB [3, 11]. Tuberculosis patients were highly prone to be infected with multiple parasites compared to TB free individuals (OR = 2.59; 95% CI: 1.90, 3.55).

Even though TB is a global problem it mainly affects sub-Saharan African countries including Ethiopia [1]. The main reason for the resurgence of TB in Africa is the link between TB and *HIV* in addition to the lack of adequate economic and human resources [2]. With the assumption of an increased risk of TB among *HIV* positive patients who co-infected with intestinal parasites, we compared TB patients by their *HIV* serostatus. However, based on the pooled analysis, a statistically significant difference was not found among *HIV* positive and *HIV* negative TB patients (OR = 0.97, 95% CI = 0.71–1.32).

### Limitation of the study

We were unable to get studies from all parts of the country, and most of the studies concentrated in the Amhara region that might not represent the whole country. Articles published other than the English language, with abstract only and incomplete information were not included. Publication bias was observed among the included studies (Fig. 2).

## Conclusion

Based on the pooled analysis, one-third of tuberculosis patients are co-infected with one or more intestinal parasites. Among the parasites, *Ascaris lumbricoides, Hookworm, Giardia lamblia,* and *Strongyloides sterocoralis* were predominantly identified. In comparison with TB free individuals, TB patients are more likely to be infected with intestinal helminths and multiple intestinal parasitic infections. Thus, we recommend routine screening of tuberculosis patients for intestinal parasites. The effect of mass deworming on tuberculosis incidence would be important to be considered in future researches.

## Supplementary information

**Additional file 1.**

**Additional file 2.**

**Additional file 3.**

## Data Availability

All relevant data are available from the corresponding author.

## Abbreviations

*HIV*: *Human Immunodeficiency Virus*
JBI: Joanna Briggs Institute Critical Appraisal
PICOS: Participants, Interventions, Comparison, Outcome, Study setting
*PRISMA*: Preferred Reporting Items for Systematic Reviews and Meta-Analyses
PROSPERO: Prospective Register of Systematic Reviews
Th: T-helper cell
Treg: T-regulatory
TST: Tuberculin Skin Test
TB: Tuberculosis

## References

[1] WHO (2019). Global Tuberculosis Report.

[2] Alene KA, Nega A, Taye BW (2013). Incidence and predictors of tuberculosis among adult people living with human immunodeficiency virus at the University of Gondar Referral Hospital, Northwest Ethiopia. BMC Infect Dis.

[3] Elias D, Mengistu G, Akuffo H, Britton S (2006). Are intestinal helminths risk factors for developing active tuberculosis?. Tropical Med Int Health.

[4] Abate E, Belayneh M, Gelaw A, Idh J, Getachew A, Alemu S (2012). The impact of asymptomatic helminth co-infection in patients with newly diagnosed tuberculosis in north-West Ethiopia. PLoS One.

[5] Neto MS, Totino RP, Sant’Anna MF, Coelho OV, Rolla CV, Zanini MG (2009). Enteroparasitosis prevalence and parasitism influence in clinical outcomes of tuberculosis patients with or without HIV co-infection in a reference Hospital in Rio de Janeiro (2000-2006). Braz J Infect Dis.

[6] Mhimbira F, Hella J, Said K, Kamwela L, Sasamalo M, Maroa T (2017). Prevalence and clinical relevance of helminth co-infections among tuberculosis patients in urban Tanzania. PLoS Negl Trop Dis.

[7] Kamal SM, El Sayed Khalifa K (2006). Immune modulation by helminthic infections: worms and viral infections. Parasite Immunol.

[8] Babu S, Nutman TB (2016). Helminth-tuberculosis co-infection: an immunologic perspective. Trends Immunol.

[9] Abate E, Belayneh M, Idh J, Diro E, Elias D, Britton S (2015). Asymptomatic Helminth infection in active tuberculosis is associated with increased regulatory and Th-2 responses and a lower sputum smear positivity. PLoS Negl Trop Dis.

[10] Tristão-Sá R, Ribeiro-Rodrigues R, Johnson LT, Pereira FEL, Dietze R (2002). Intestinal nematodes and pulmonary tuberculosis. Rev Soc Bras Med Trop.

[11] Alemu A, Kebede A, Dagne B, Amare M, Diriba G, Yenew B (2019). Intestinal parasites co-infection and associated factors among active pulmonary tuberculosis patients in selected health centers, Addis Ababa, Ethiopia: unmatched case control study. BMC Infect Dis.

[12] Hailu AW, Merid Y, Gebru AA, Ayene YY, Asefa MK (2015). The case control studies of HIV and intestinal parasitic infections rate in active pulmonary tuberculosis patients in Woldia General Hospital and Health Center in North Wollo, Amhara Region, Ethiopia. Int J Pharma Sci.

[13] Wegayehu T, Tsalla T, Seifu B, Teklu T (2013). Prevalence of intestinal parasitic infections among highland and lowland dwellers in Gamo area, South Ethiopa. BMC Public Health.

[14] Feleke BE, Feleke TE, Biadglegne F (2019). Nutritional status of tuberculosis patients, a comparative cross-sectional study. BMC Pulm Med.

[15] Alemayehu M, Birhan W, Belyhun Y, Sahle M, Tessema B (2014). Prevalence of smear positive tuberculosis, intestinal parasites and their co-infection among tuberculosis suspects in Gondar University hospital and Gondar poly clinic, north West Ethiopia. J Microbial Biochem Technol.

[16] Ramos JM, Reyes F, Tesfamariam A (2006). Intestinal parasites in adults admitted to a rural ethiopian hospital: relationship to tuberculosis and malaria. Scand J Infect Dis.

[17] Tegegne Y, Wondmagegn T, Worku L, Jejaw Zeleke A (2018). Prevalence of intestinal parasites and associated factors among pulmonary tuberculosis suspected patients attending University of Gondar Hospital, Gondar, Northwest Ethiopia. J Parasitol Res.

[18] Munn Z, Aromataris E, Tufanaru C, Stern C, Porritt K, Farrow J (2019). The development of software to support multiple systematic review types: the Joanna Briggs institute system for the unified management, assessment and review of information (JBI SUMARI). Int J Evid Based Healthc.

[19] Getaneh A, Mama M (2017). Intestinal helminth co-infection and associated factors among tuberculosis patients in Arba Minch, Ethiopa. BMC Infect Dis.

[20] Kassu A, Mengistu G, Ayele B, Diro E, Mekonnen F, Ketema D (2007). HIV and intestinal parasites in adult TB patients in a teaching hospital in Northwest Ethiopia. Trop Dr.

[21] Gashaw F, Bekele S, Mekonnen Y, Medhin G, Ameni G, Erko B (2019). High helminthic co-infection in tuberculosis patients with undernutritional status in northeastern Ethiopia. Infect Dis Poverty.

[22] Porritt K, Gomersall J, Lockwood C (2014). JBI’s systematic reviews: study selection and critical appraisal. Am J Nurs.

[23] Sterne JA, Egger M (2001). Funnel plots for detecting bias in meta-analysis: guidelines on choice of axis. J Clin Epidemiol.

[24] Sterne JA, Egger M (2005). Regression methods to detect publication and other bias in meta-analysis. Publication bias in meta-analysis: Prevention, assessment and adjustments.

[25] RevMan R (2014). The nordic cochrane centre, the cochrane collaboration. Book [computer program].

[26] Taye B, Desta K, Ejigu S, Dori GU (2014). The magnitude and risk factors of intestinal parasitic infection in relation to human immunodeficiency virus infection and immune status, at ALERT hospital, Addis Ababa, Ethiopa. Parasitol Int.

[27] Gebreegziabiher D, Desta K, Desalegn G, Howe R, Abebe M (2014). The effect of maternal helminth infection on maternal and neonatal immune function and immunity to tuberculosis. PLoS One.

[28] Amare B, Moges B, Mulu A, Yifru S, Kassu A (2015). Quadruple burden of HIV/AIDS, tuberculosis, chronic intestinal parasitoses, and multiple micronutrient deficiency in Ethiopia: a summary of available findings. Biomed Res Int.

[29] Elias D, Wolday D, Akuffo H, Petros B, Bronneru BS (2001). Effect of deworming on human T cell responses to mycobacterial antigens in helminth-exposed individuals before and after Bacille Calmette GueÂrin (BCG) vaccination. Clin Exp Immunol.

[30] Gelaw A, Abate E, Idh J, Mulu A, Anagaw B, Belyhun Y (2012). Plasma IgE level and eosinophil count in smear positive tuberculosis patients with and without helminthic infections at Gondar University Hospital, Northwest Ethiopia. Eur J Exp Biol.

[31] Kassu A, Mohammad A, Fujimaki Y, Moges F, Elias D, Mekonnen F (2004). Serum IgE levels of tuberculosis patients in a tropical setup with high prevalence of HIV and intestinal parasitoses. Clin Exp Immunol.

[32] Abate E, Elias D, Getachew A, Alemu S, Diro E, Britton S (2015). Effects of albendazole on the clinical outcome and immunological responses in helminth co-infected tuberculosis patients: a double blind randomised clinical trial. Int J Parasitol.

[33] Kassu A, Fujino M, Nishizawa M, Mengistu G, Diro E (2008). Levels of serum HIV-1 RNA viral load in tuberculosis patients with or without intestinal parasites during treatment of tuberculosis in Gondar, Ethiopia. Ethiop J Health Biomed Sci.

[34] Taghipour A, Azimi T, Javanmard E, Pormohammad A, Olfatifar M, Rostami A (2018). Immunocompromised patients with pulmonary tuberculosis; a susceptible group to intestinal parasites. Gastroenterol Hepatol Bed Bench.

[35] Li XX, Chen JX, Wang LX, Tian LG, Zhang YP, Dong SP (2014). Intestinal parasite co-infection among pulmonary tuberculosis cases without human immunodeficiency virus infection in a rural county in China. Am J Trop Med Hyg.

[36] Hasanain AF, Zayed AA, Mahdy RE, Nafee AM, Attia RA, Mohamed AO (2015). Hookworm infection among patients with pulmonary tuberculosis: impact of co-infection on the therapeutic failure of pulmonary tuberculosis. Int J Mycobacteriol.

