# Supplementary figures and images for: Intestinal parasites co-infection among tuberculosis patients in Ethiopia: a systematic review and meta-analysis
Source: BMC Infect Dis. 2020 Jul 14;20:510. doi: 10.1186/s12879-020-05237-7 (PMC7362415; doi:10.1186/s12879-020-05237-7)

Search strings

Pubmed


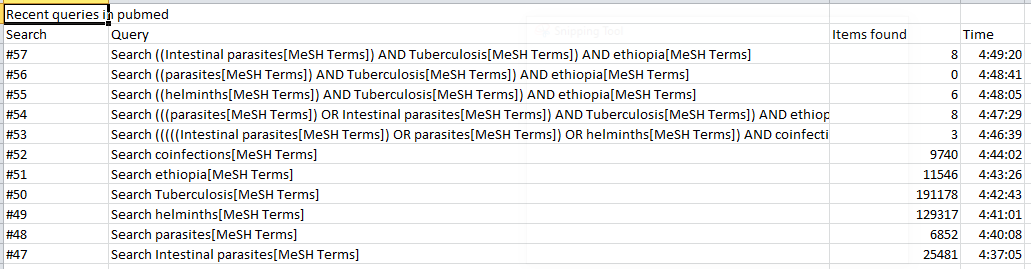


Medline


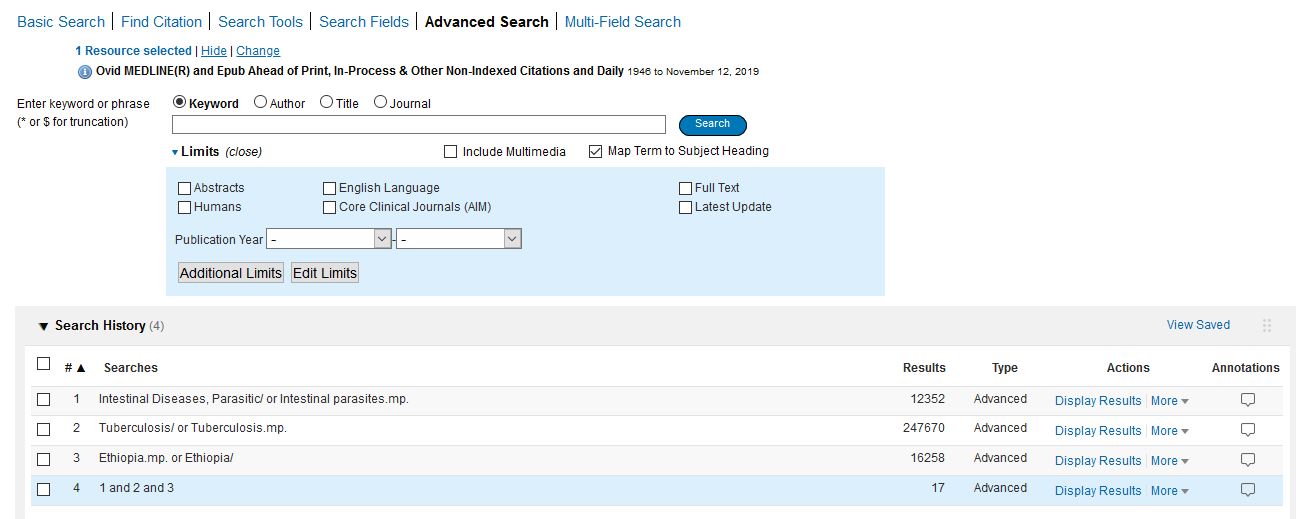


Embase


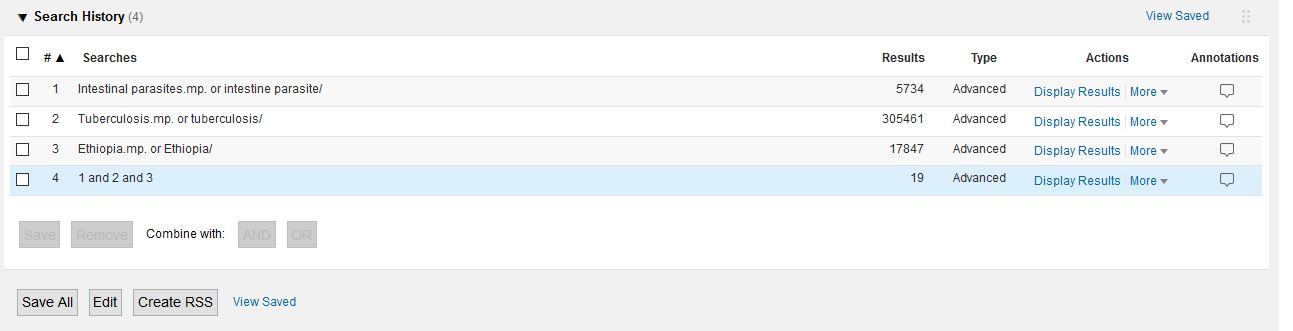


Emcare


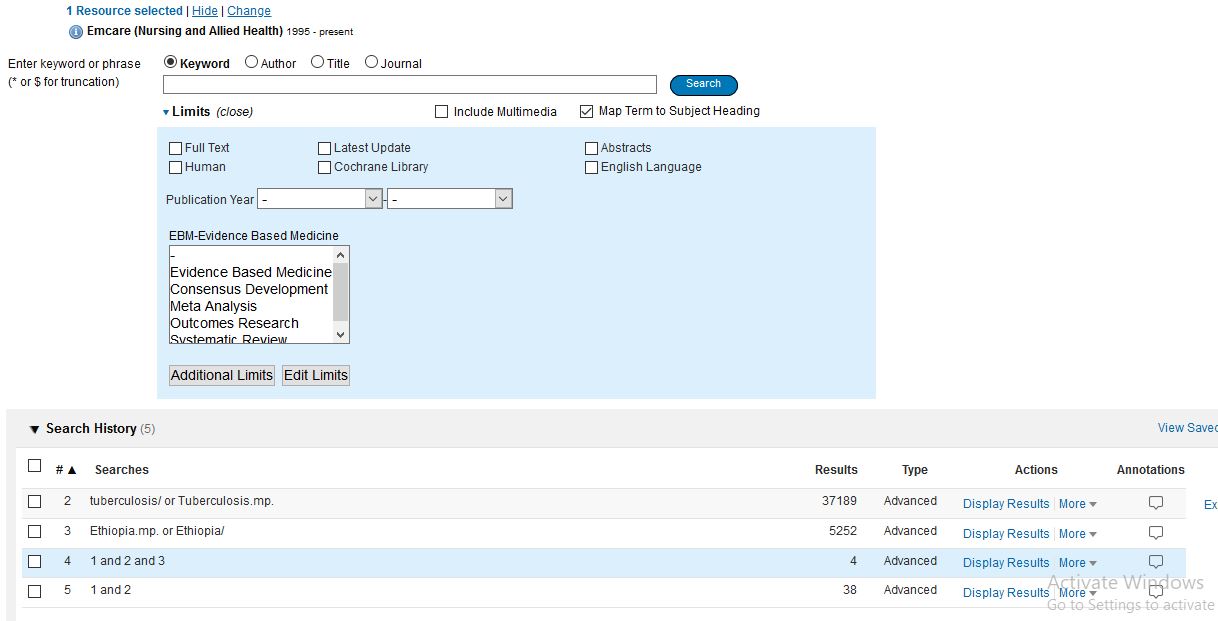


CINAHL


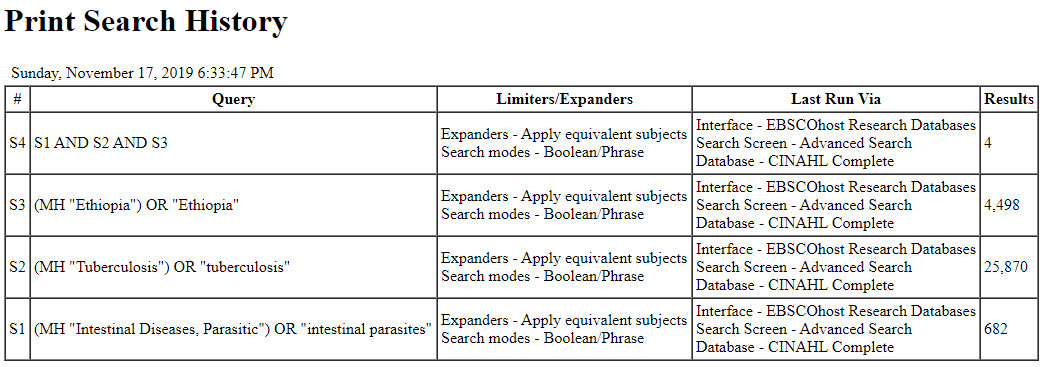


DOAJ


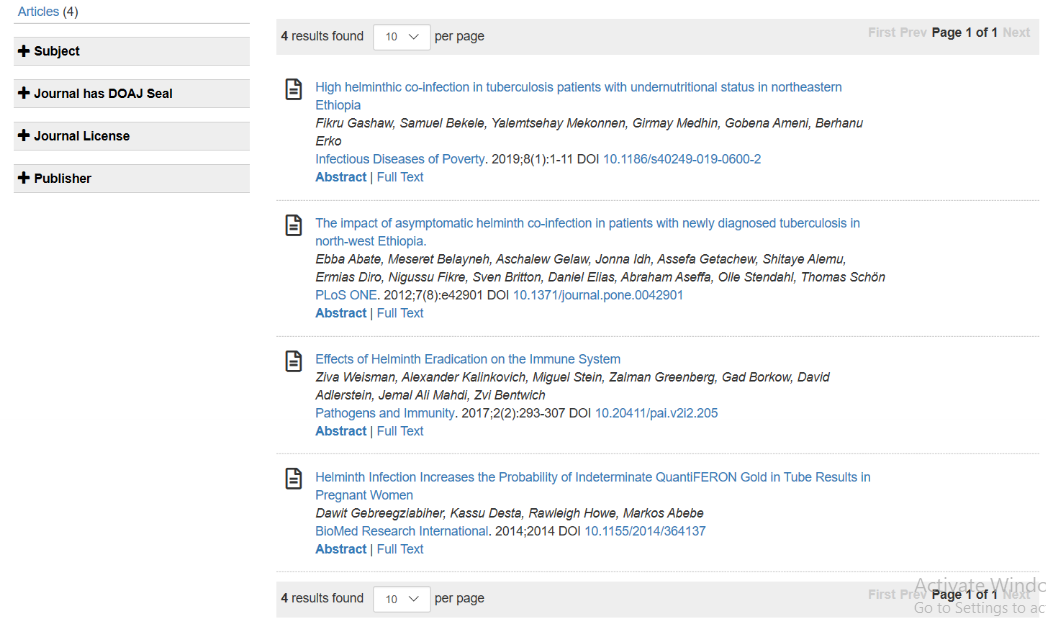

Supplement: Supplementary file 1 — Additional file 1. [file 12879_2020_5237_MOESM1_ESM.docx]
