# Supplementary material for: Intestinal parasites co-infection among tuberculosis patients in Ethiopia: a systematic review and meta-analysis
Source: BMC Infect Dis. 2020 Jul 14;20:510. doi: 10.1186/s12879-020-05237-7 (PMC7362415; doi:10.1186/s12879-020-05237-7)
Supplement: Supplementary file 2 — Additional file 2. [file 12879_2020_5237_MOESM2_ESM.docx]

Critical appraisal for case control studies

| Author, year | Q1 | | | | Q2 | | | | Q3 | | | | Q4 | | | | Q5 | | | | Q6 | | | | Q7 | | | | Q8 | | | | Q9 | | | | Q10 | | | | Overall quality result |
| --- | --- | --- | --- | --- | --- | --- | --- | --- | --- | --- | --- | --- | --- | --- | --- | --- | --- | --- | --- | --- | --- | --- | --- | --- | --- | --- | --- | --- | --- | --- | --- | --- | --- | --- | --- | --- | --- | --- | --- | --- | --- |
|  | Y | N | U | NA | Y | N | U | NA | Y | N | U | NA | Y | N | U | NA | Y | N | U | NA | Y | N | U | NA | Y | N | U | NA | Y | N | U | NA | Y | N | U | NA | Y | N | U | NA |  |
| Alemu *et al*, 2019 | √ |  |  |  | √ |  |  |  | √ |  |  |  | √ |  |  |  | √ |  |  |  |  | √ |  |  |  | √ |  |  | √ |  |  |  | √ |  |  |  | √ |  |  |  | 8/10 (80%) |
| Hailu *et a*l, 2015 | √ |  |  |  | √ |  |  |  | √ |  |  |  | √ |  |  |  | √ |  |  |  | √ |  |  |  | √ |  |  |  | √ |  |  |  | √ |  |  |  | √ |  |  |  | 10/10 (100%) |
| Elias *et al*, 2006 | √ |  |  |  | √ |  |  |  | √ |  |  |  | √ |  |  |  | √ |  |  |  | √ |  |  |  | √ |  |  |  | √ |  |  |  | √ |  |  |  | √ |  |  |  | 10/10 (100%) |
| Abate *et al,* 2012 | √ |  |  |  | √ |  |  |  | √ |  |  |  | √ |  |  |  | √ |  |  |  |  |  | √ |  |  |  | √ |  | √ |  |  |  | √ |  |  |  | √ |  |  |  | 8/10 (80%) |

****Y=yes, N=no, U=unclear, NA=not applicable, <60%=low, 60-80%=medium, >80%=high quality***

Critical appraisal for cross sectional studies

| Author, year | Q1 | | | | Q2 | | | | Q3 | | | | Q4 | | | | Q5 | | | | Q6 | | | | Q7 | | | | Q8 | | | | Overall quality result | |
| --- | --- | --- | --- | --- | --- | --- | --- | --- | --- | --- | --- | --- | --- | --- | --- | --- | --- | --- | --- | --- | --- | --- | --- | --- | --- | --- | --- | --- | --- | --- | --- | --- | --- | --- |
|  | Y | N | U | NA | Y | N | U | NA | Y | N | U | NA | Y | N | U | NA | Y | N | U | NA | Y | N | U | NA | Y | N | U | NA | Y | N | U | NA |  | |
| Alemu *et al*, 2017 | √ |  |  |  | √ |  |  |  | √ |  |  |  | √ |  |  |  | √ |  |  |  | √ |  |  |  | √ |  |  |  | √ |  |  |  | 8/8 (100%) | |
| Kassu *et al*, 2007 | √ |  |  |  | √ |  |  |  | √ |  |  |  | √ |  |  |  |  |  | √ |  |  |  | √ |  | √ |  |  |  | √ |  |  |  | 6/8 (75%) |  |
| Alemayehu *et al*, 2014 | √ |  |  |  | √ |  |  |  | √ |  |  |  | √ |  |  |  |  | √ |  |  | √ |  |  |  | √ |  |  |  | √ |  |  |  | 6/8 (75%) |  |
| Tegegne *et al*, 2018 | √ |  |  |  | √ |  |  |  | √ |  |  |  | √ |  |  |  |  |  | √ |  |  |  | √ |  | √ |  |  |  | √ |  |  |  | 6/8 (75%) |  |
| Gashaw *et al*, 2019 | √ |  |  |  | √ |  |  |  | √ |  |  |  | √ |  |  |  |  |  | √ |  |  |  | √ |  | √ |  |  |  | √ |  |  |  | 6/8 (75%) |  |
| Ramose *et al*, 2009 | √ |  |  |  | √ |  |  |  |  |  | √ |  | √ |  |  |  |  |  | √ |  |  |  | √ |  | √ |  |  |  | √ |  |  |  | 5/8 (62.5%) |  |
| Feleke *et al*, 2019 | √ |  |  |  | √ |  |  |  | √ |  |  |  | √ |  |  |  |  |  | √ |  |  |  | √ |  | √ |  |  |  | √ |  |  |  | 6/8 (75%) |  |

****Y=yes, N=no, U=unclear, NA=not applicable, <60%=low, 60-80%=medium, >80%=high quality***
