# Supplementary material for: Intestinal parasites co-infection among tuberculosis patients in Ethiopia: a systematic review and meta-analysis
Source: BMC Infect Dis. 2020 Jul 14;20:510. doi: 10.1186/s12879-020-05237-7 (PMC7362415; doi:10.1186/s12879-020-05237-7)
Supplement: Supplementary file 3 — Additional file 3. [file 12879_2020_5237_MOESM3_ESM.docx]

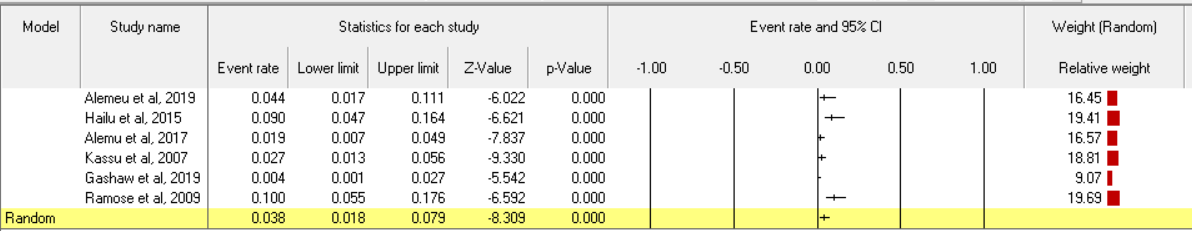
*E. Histolytica*


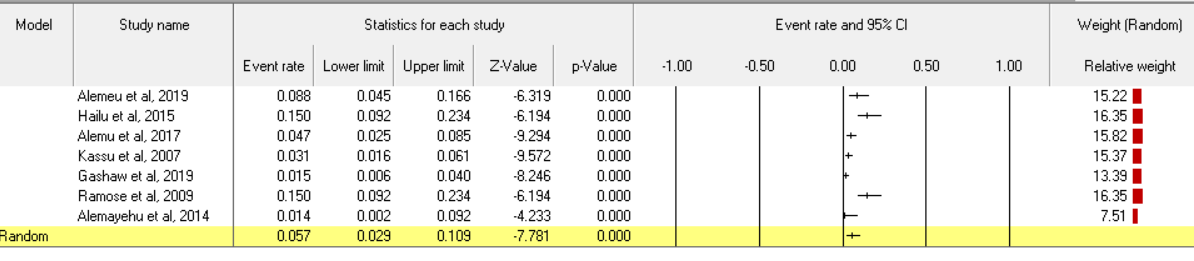
*G. lamblia*


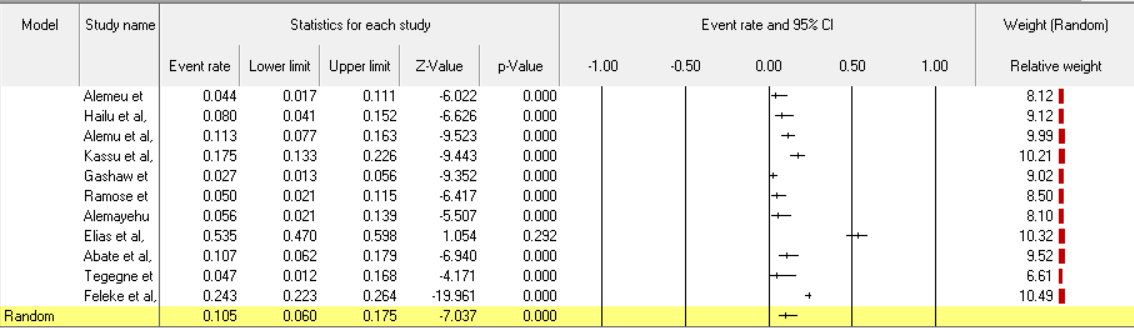
*A.lumbericoids*


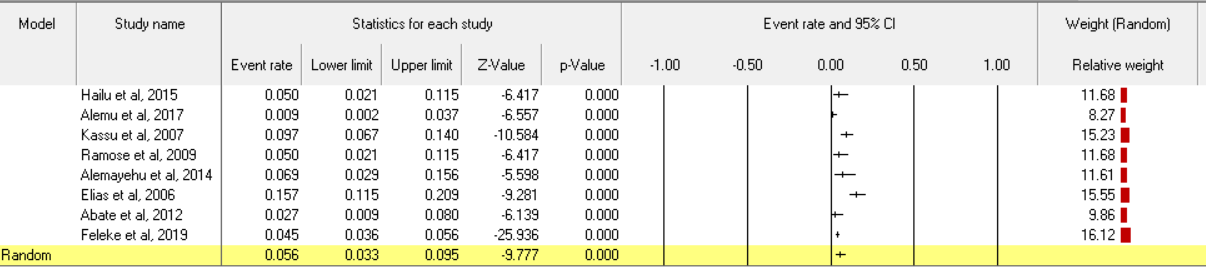
*S.sterocoralis*


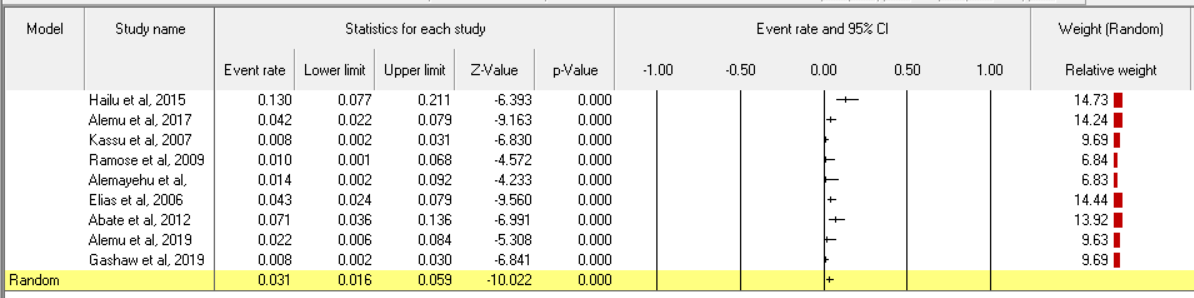
*T.tricuria*


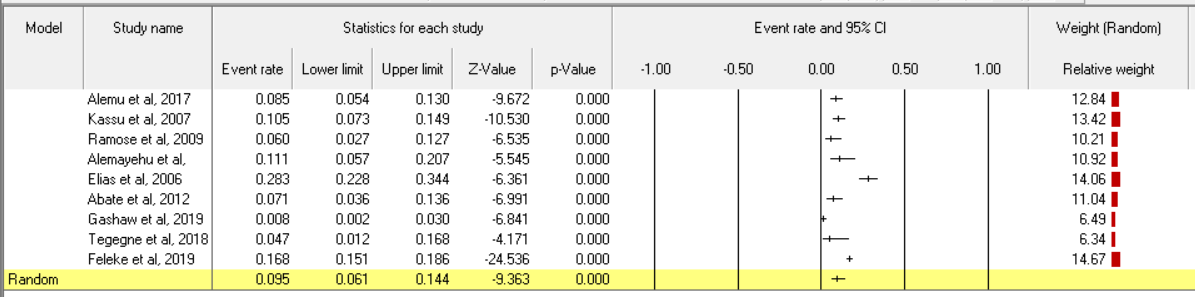
*Hookworm*


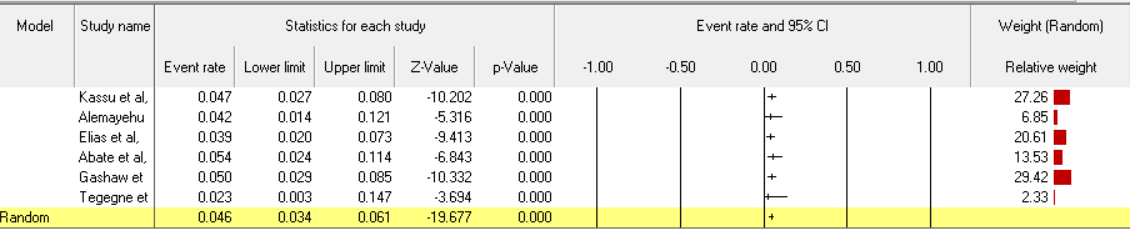
*S.mansoni*


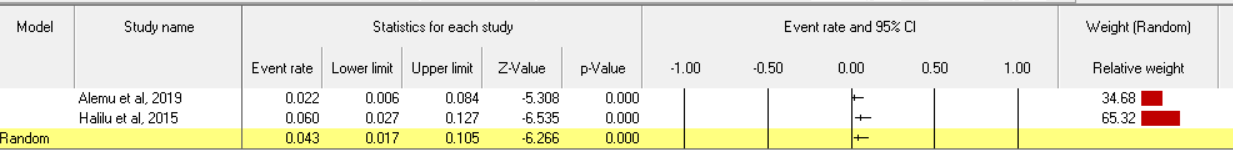
*C.parvum*


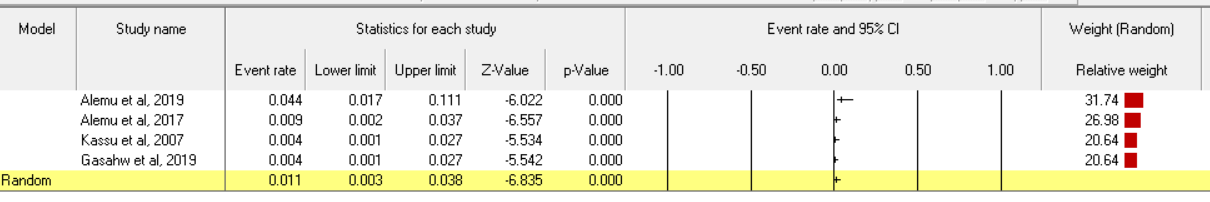
*H.nana*


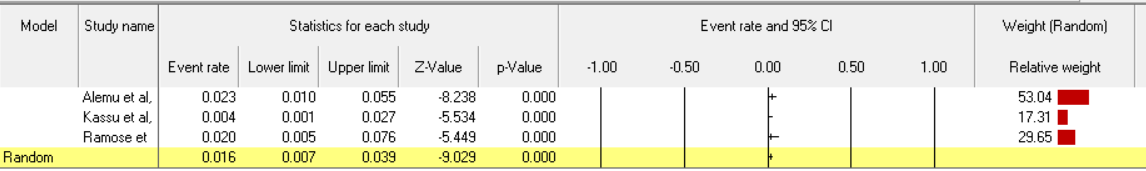


*Taenia species*


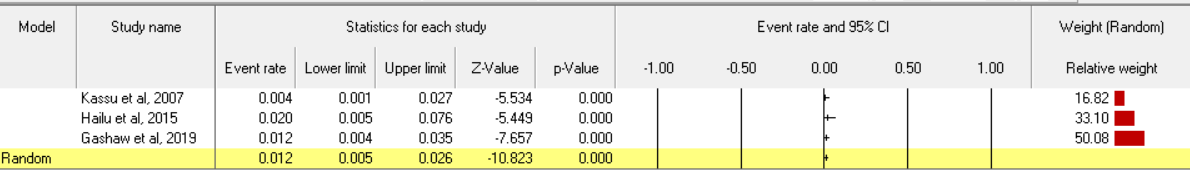
*E.vermicularies*

**Supplementary figures:** Pooled prevalence of different intestinal parasites among tuberculosis patients in Ethiopia.
